# Engineered SARS-CoV-2 receptor binding domain improves manufacturability in yeast and immunogenicity in mice

**DOI:** 10.1073/pnas.2106845118

**Published:** 2021-09-07

**Authors:** Neil C. Dalvie, Sergio A. Rodriguez-Aponte, Brittany L. Hartwell, Lisa H. Tostanoski, Andrew M. Biedermann, Laura E. Crowell, Kawaljit Kaur, Ozan S. Kumru, Lauren Carter, Jingyou Yu, Aiquan Chang, Katherine McMahan, Thomas Courant, Celia Lebas, Ashley A. Lemnios, Kristen A. Rodrigues, Murillo Silva, Ryan S. Johnston, Christopher A. Naranjo, Mary Kate Tracey, Joseph R. Brady, Charles A. Whittaker, Dongsoo Yun, Natalie Brunette, Jing Yang Wang, Carl Walkey, Brooke Fiala, Swagata Kar, Maciel Porto, Megan Lok, Hanne Andersen, Mark G. Lewis, Kerry R. Love, Danielle L. Camp, Judith Maxwell Silverman, Harry Kleanthous, Sangeeta B. Joshi, David B. Volkin, Patrice M. Dubois, Nicolas Collin, Neil P. King, Dan H. Barouch, Darrell J. Irvine, J. Christopher Love

**Affiliations:** ^a^Department of Chemical Engineering, Massachusetts Institute of Technology (MIT), Cambridge, MA 02139;; ^b^The Koch Institute for Integrative Cancer Research, Massachusetts Institute of Technology, Cambridge, MA 02139;; ^c^Department of Biological Engineering, Massachusetts Institute of Technology, Cambridge, MA 02139;; ^d^Ragon Institute of Massachusetts General Hospital (MGH), MIT, Harvard, Cambridge, MA 02139;; ^e^Center for Virology and Vaccine Research, Beth Israel Deaconess Medical Center, Harvard Medical School, Boston, MA 02115;; ^f^Department of Pharmaceutical Chemistry, Vaccine Analytics, and Formulation Center, University of Kansas, Lawrence, KS 66047;; ^g^Department of Biochemistry, University of Washington, Seattle, WA 98195;; ^h^Institute for Protein Design, University of Washington, Seattle, WA 98195;; ^i^Vaccine Formulation Institute, 1228 Plan-Les-Ouates, Geneva, Switzerland;; ^j^Harvard-MIT Health Sciences and Technology, Institute for Medical Engineering and Science, Massachusetts Institute of Technology, Cambridge, MA 02139;; ^k^Bioqual, Inc., Rockville, MD 20850;; ^l^Bill & Melinda Gates Medical Research Institute, Cambridge, MA 02139;; ^m^Bill & Melinda Gates Foundation, Seattle, WA 98109;; ^n^Massachusetts Consortium on Pathogen Readiness, Boston, MA 02115;; ^o^Howard Hughes Medical Institute, Chevy Chase, MD 20815

**Keywords:** protein vaccine, *Pichia pastoris*, SARS-CoV-2, manufacturability

## Abstract

Global containment of COVID-19 still requires accessible and affordable vaccines for low- and middle-income countries (LMICs). Recently approved vaccines provide needed interventions, albeit at prices that may limit their global access. Subunit vaccines based on recombinant proteins are suited for large-volume microbial manufacturing to yield billions of doses annually, minimizing their manufacturing cost. These types of vaccines are well-established, proven interventions with multiple safe and efficacious commercial examples. Many vaccine candidates of this type for SARS-CoV-2 rely on sequences containing the receptor-binding domain (RBD), which mediates viral entry to cells via ACE2. Here we report an engineered sequence variant of RBD that exhibits high-yield manufacturability, high-affinity binding to ACE2, and enhanced immunogenicity after a single dose in mice compared to the Wuhan-Hu-1 variant used in current vaccines. Antibodies raised against the engineered protein exhibited heterotypic binding to the RBD from two recently reported SARS-CoV-2 variants of concern (501Y.V1/V2). Presentation of the engineered RBD on a designed virus-like particle (VLP) also reduced weight loss in hamsters upon viral challenge.

## Significance

Most of the global population resides in low- and middle-income countries, where current vaccines for COVID-19 remain largely unavailable. For the COVID-19 pandemic, the world will need access to >10 billion doses of vaccines, or more than double the annual volume of vaccines for all other diseases. Many vaccine candidates use the SARS-CoV-2 receptor-binding domain (RBD) antigen. Here, we present an engineered RBD with improved production titers in *Pichia pastoris*, a yeast commonly used for large-scale, low-cost manufacturing by vaccine manufacturers. The modified RBD also raises an enhanced immune response in mice relative to the Wuhan-Hu-1 sequence used in current candidates. These combined traits make it a promising candidate for next-generation vaccines addressing emerging variants of the virus.

Prevention of COVID-19 on a global scale will require >10 billion doses of vaccines for SARS-CoV-2; most of which are needed in low- and middle-income countries (LMICs) (1). To ensure adequate supply and global access, vaccine manufacturers must select highly immunogenic vaccine antigens that offer broad protection against emerging variants and are compatible with large-volume production in existing manufacturing facilities (2, 3). Vaccines using mRNA have established the efficacy of vaccines for SARS-CoV-2 based on full-length trimeric spike (S) protein (4, 5). Recombinant S protein produced in mammalian or insect cells has also shown immunogenicity and efficacy in nonhuman primates (6). Protein-based vaccines hold promise for large-volume, low-cost production, and are safe and efficacious (7–9). The receptor-binding domain (RBD) subunit, which mediates viral entry to cells via ACE2 (10–12), has emerged as an important alternative antigen (13). Antibodies to RBD account for most of the neutralizing activity elicited in natural infections, and several potent monoclonal antibodies have been discovered from convalescent patients (14, 15). A His-tagged SARS-CoV-2 RBD construct based on SARS-CoV-2 Wuhan-Hu-1 and produced in insect cells has elicited neutralizing antibodies in mice and protective immunity in nonhuman primates (16). Similar tagged constructs have also been adapted for production in yeast like *Komagataella* *phaffii* (*Pichia pastoris*) (17, 18), establishing the RBD domain as a prominent candidate for large-volume manufacturing of COVID-19 vaccines.

Despite its significance for low-cost vaccine candidates, recombinant RBD based on the original SARS-CoV-2 clade 19A sequence has shown limited immunogenicity to date. Reported candidates would require as many as three doses or large doses to elicit strong neutralizing antibody responses in mice when formulated with adjuvants (16, 18). Increasing the number of doses or amounts required could limit its benefits for affordable and accessible vaccines. An engineered design for the RBD, therefore, could enhance the potency of many subunit-based vaccine candidates using this domain.

## Results

We reasoned that an improved RBD variant for vaccine candidates should exhibit both improved quality attributes relevant for manufacturing (increased titers, reduced aggregation) and immunogenicity relative to the Wuhan-Hu-1 sequence used in current vaccines. We further sought to develop a variant amenable to production in microbial hosts, which can be cultured at very large volumes (up to 50,000+ liters) and low costs. Based on previous reports for similar constructs from SARS-CoV-1 and Middle East Respiratory Syndrome (MERS)–CoV with demonstrated immunogenicity (13, 19, 20), we first chose to evaluate the production of a tagless 201 amino acid sequence within the RBD (S protein amino acids 332 to 532) by secretion from yeast (*SI Appendix*, Fig. S1*A*).

**Fig. 1.**
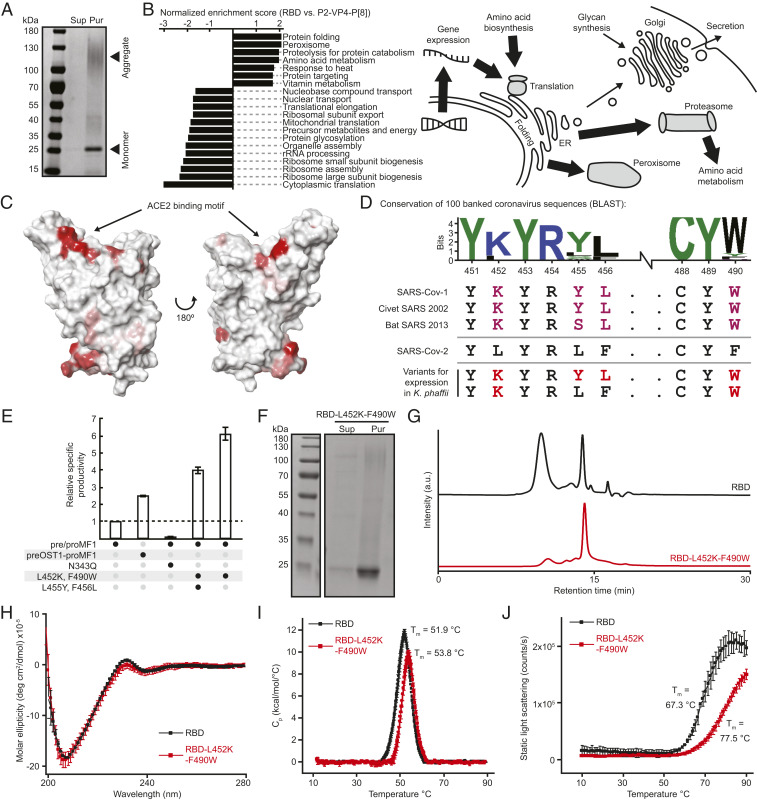
Molecular engineering of the RBD for manufacturability. (*A*) Reduced SDS-PAGE of purified RBD. Sup, cultivation supernatant; Pur, purified protein. (*B*) Gene set enrichment analysis comparing strains expressing RBD and a rotavirus VP8 fragment (*Left*); schematic model based on pathways for degradation of the RBD in the proteasome and peroxisome, with higher flux of recombinant protein shown with larger arrows (*Right*). (*C*) Structural rendering of RBD (predicted hydrophobic patches are red). (*D*) Sequence logo of predicted ACE2 binding motif hydrophobic patch using the top 96 sequences homologous to SARS-CoV-2. Alignment of the ACE2 binding motif to other sarbecoviruses, including selected designs for testing. (*E*) Bar graph of relative specific productivity for engineered variants of the RBD. preOST1-proMF1 is an alternative signal peptide. Reported values are relative to expression of wild-type RBD. (*F*) Reduced SDS-PAGE of purified RBD-L452K-F490W. (*G*) Size exclusion chromatography of purified RBD variants. (*H*) Far-UV circular dichroism at 10 °C of purified RBD variants. (*I*) Differential scanning calorimetry of purified RBD variants. (*J*) Static light scattering vs. temperature of purified RBD variants.

We created a two-stage chromatographic method to purify RBD based on its biophysical characteristics and prior experience purifying heterologous proteins with similar molecular weight, isoelectric point, and hydrophobicity (21–23). We produced RBD in a 200-mL shake flask culture and purified quantities to assess the quality attributes of the protein (Fig. 1*A*). The resulting protein bound human ACE2-Fc (K_D_ 49 ± 22 nM) and CR3022 (a neutralizing antibody to SARS-CoV-1 with cross-reactivity to SARS-CoV-2) (K_D_ 32 ± 2 nM) (*SI Appendix*, Fig. S1 *B* and *C*) (24). The protein displayed high mannose glycoforms at the single canonical position for N-linked glycosylation present on the exposed surface distal from the receptor binding motif (RBM) (*SI Appendix*, Fig. S1*D*). The protein, however, exhibited a strong tendency to form high-molecular-weight species (evident in sodium dodecyl sulfate–polyacrylamide gel electrophoresis [SDS-PAGE] and size exclusion chromatography [SEC]), particularly in fermentations with high air–water interfaces. The titers were also limited (∼12 mg/L), similar to previously described titers and product quality of unoptimized fermentation (25). Together, these results suggested production of this domain was feasible, but presented concerns regarding potential yields and consistency for large-volume manufacturing.

From these assessments, we reasoned that the qualities of the protein itself may impede its expression and ultimately its attributes that would influence its suitability as an immunogen for subunit-based vaccine candidates. We hypothesized that the tendency of the protein to self-associate may also induce stress on the host cells during the expression and secretion of the protein. Efficient secretion of recombinant protein by yeast requires successful folding and modification of the nascent peptide in the endoplasmic reticulum (ER) (26). Insoluble or misfolded protein inside the host cells could lead to an unfolded protein response and subsequent degradation of the recombinant product, reducing its production. To further evaluate this relationship, we performed a genome-scale analysis of the yeast by RNA-sequencing and compared the gene expression to another strain capable of producing a subunit vaccine candidate for rotavirus (P2-VP4-P[8]) of similar size and complexity at commercially relevant productivities (exceeding 0.5 g/L/d) (23). This reference strain has no additional engineered genomic modifications, so we hypothesized that the observed differences in gene expression between the two strains were due to the host’s response to the two different recombinant proteins. Analysis of the differentially regulated pathways revealed differences in gene sets related to protein folding and ER-associated protein degradation pathways. These were up-regulated relative to the strain secreting P2-VP4-P[8], implying that the recombinant RBD may be routed from the ER for degradation, reducing yields (Fig. 1*B*).

Small, conservative changes to a protein sequence can address quality attributes of the protein such as aggregation and also reduce strain on cellular functions to improve titer (23). We undertook a similar approach to molecular engineering for SARS-CoV-2 RBD. We inspected the predicted folded structure of the RBD and identified several hydrophobic patches on the surface of this molecule that could promote noncovalent multimerization (Fig. 1*C*). Spike protein amino acids 452 to 456 and 488 to 490 had the highest predicted regions of hydrophobicity and are located in the ACE2 RBM (spike protein residues 437 to 507) (27). The second hydrophobic patch comprised internal residues that are typically hidden within the trimeric spike protein, but become exposed surface residues on the RBD subunit (*SI Appendix*, Fig. S1*E*). To mitigate these hydrophobic patches, we replaced hydrophobic residues with amino acids highly conserved among other sarbecoviruses known to bind ACE2 (Fig. 1*D*) (28, 29). Lysine residues (as found in other coronaviruses in this region) are generally known to influence adjacent regions by reducing the propensity for aggregation (30). Replacement of only 1 to 4 amino acids in the RBM in silico reduced the AggScore (31), a predicted metric of hydrophobicity, of the RBD from 151.26 to 132.46. Based on this analysis, we tested five variants of RBD (*SI Appendix*, Fig. S1*F*) and found two of these variants (RBD-L452K-F490W and RBD-L452K-L455Y-F456L-F490W) exhibited four- to sixfold increased specific productivity relative to the strain producing the original RBD (Fig. 1*E*) (32). We selected the RBD-L452K-F490W to characterize further since it required fewer total changes from the original Wuhan-Hu-1 sequence. We purified RBD-L452K-F490W (Fig. 1*F*) and observed by size exclusion chromatography that only 17% of the total protein formed high-molecular-weight species compared to 59% in the original purified RBD (Fig. 1*G*). We then produced and purified multiple milligrams of each antigen using our InSCyT manufacturing systems for automated, end-to-end production (*SI Appendix*, Fig. S1 *G* and *H*) (21). RBD-L452K-F490W exhibited similar secondary structure to the original wild-type (WT) sequence (Fig. 1*H*). The modified sequence manifested a higher melting temperature compared to the original RBD (Fig. 1*I*), and static light scattering as a function of temperature revealed that thermally induced aggregation of RBD-L452K-F490W was shifted nearly 10 °C higher than unmodified RBD (Fig. 1*J*).

Finally, we reassessed differences in gene expression between strains expressing RBD and RBD-L452K-F490W (*SI Appendix*, Fig. S1*I*). Contrasting the results for the strain producing the original RBD sequence, the strain expressing RBD-L452K-F490W did not up-regulate gene sets related to protein folding and ER-associated protein degradation relative to the strain expressing P2-VP4-P[8], suggesting that the L452K-F490W mutations may alleviate this source of cellular stress. These transcriptomic and biophysical data together suggest the targeted changes to reduce the hydrophobicity of residues within the RBM reduced the propensity for aggregation, enhanced the thermostability of the protein, and improved expression—these traits are all important for large-volume production as well as development of a formulated product with reduced thermal requirements for storage.

**Fig. 2.**
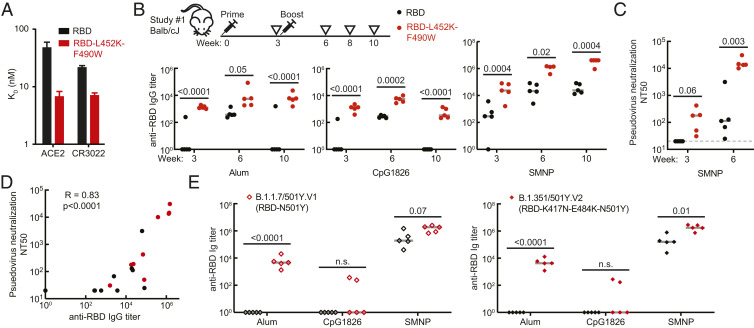
Immunogenicity and antigenicity of wild-type and engineered RBD with single adjuvants. (*A*) Binding of purified RBD variants to human ACE2–IgG fusion protein and CR3022 neutralizing antibody by biolayer interferometry. (*B*) Titer of RBD-specific IgG in mouse sera by ELISA. Gray lines represent median values. (*C*) Titer of neutralizing antibody in mouse sera from SARS-CoV-2 pseudovirus neutralization assay. (*D*) Correlation of anti-RBD IgG ELISA and pseudovirus neutralization from mouse sera with SMNP adjuvant. (*E*) Titer of RBD-specific IgG in week 8 mouse sera from mice inoculated with RBD or RBD-L452K-F490W, evaluated for binding against RBD proteins with mutations from circulating strains of SARS-CoV-2. Data points represent individual animals. Gray lines represent median values. Significance was determined by *t* test, with Holm–Sidak correction. *P* values are indicated on plots. LOD, limit of detection.

The L452K and F490W mutations were selected from conserved substitutions identified from other sarbecoviruses and improved the quality attributes of the RBD, but these changes could alter the antigenicity and immunogenicity of the molecule. Several identified neutralizing antibodies from patients recognize epitopes around the RBM, and many bind near L452 (33). We measured the affinities of the RBD variant to both human ACE2-Fc and CR3022, and surprisingly, the RBD-L452K-F490W exhibited higher binding affinity to both molecules (K_D_ = 7 ± 1 and 7 ± 1 nM) (Fig. 2*A*). These data confirmed the engineered RBD variant retains its antigenicity relative to the Wuhan-Hu-1 sequence.

To assess the immunogenicity of our engineered variant, we subcutaneously immunized Balb/cJ mice with 5 µg of the unmodified RBD (332 to 532) or RBD-L452K-F490W formulated with different adjuvants (a saponin-based adjuvant [SMNP], aluminum hydroxide referred to as alum, or CpG1826) and boosted mice with an identical dose 21 d later. All animals immunized with RBD-L452K-F490W seroconverted after a single dose (Fig. 2*B*). Anti-RBD IgG titers remained consistently elevated over 7 wk postboost with alum and SMNP adjuvants, while we observed a 10-fold decrease in titer with CpG adjuvant (*SI Appendix*, Fig. S2*A*). In contrast, anti-RBD IgG responses in animals immunized with unmodified Wuhan-Hu-1 RBD were significantly less robust and less durable; serum titers from mice receiving the wild-type sequence with either alum or CpG declined to basal levels over time. Furthermore, immunization with RBD-L452K-F490W with SMNP elicited pseudovirus neutralizing antibody (NAb) titers after only one dose, with NT50 titers exceeding 10^4^ after a second dose (Fig. 2 *C* and *D*). These NAb levels were significantly greater than those elicited by the WT sequence both postprime and postboost. For comparison, we previously reported NAb titers from human convalescent sera between 10^2^ and 10^3^ using the same pseudovirus neutralization assay (34, 35).

We also evaluated subtype biases in the immune response. Of note, the SMNP adjuvant elicited anti-RBD IgG across a distribution of isotypes, including isotypes associated with Th1 (IgG2a and IgG2b) and Th2 (IgG1) responses (*SI Appendix*, Fig. S2*B*). We calculated the ratio of IgG2 antibodies to total IgG antibodies and observed less IgG2 bias in animals immunized with RBD-L452K-F490W and SMNP (*SI Appendix*, Fig. S2*C*). Animals immunized with RBD-L452K-F490W and alum exhibited an IgG1-dominant response, consistent with a strong Th2 bias. These results demonstrate the potential of RBD-L452K-F490W to elicit an immune response in mice with only a single adjuvant.

Other adjuvants, including MF59, Matrix-M, and aluminum salts have previously been shown to promote functional neutralizing responses for SARS-CoV-1 and MERS (36). In a second study to examine other potential adjuvants, we immunized C57BL/6J mice with either 2 µg or 10 µg of the RBD-L452K-F490W protein and boosted mice 21 d later. The RBD-L452K-F490W immunogen also elicited seroconversion in mice similar to full-length S protein when used in combination with oil-in-water emulsion or liposome-based adjuvants (*SI Appendix*, Fig. S2*D*). In this study, we observed a bias toward IgG2 that varied with adjuvant, suggesting that choice of adjuvant may influence the type of immune response mediated in mice (*SI Appendix*, Fig. S2*E*). Together, these results indicate the engineered variant exhibits enhanced immunogenicity superior to the Wuhan-Hu-1 RBD sequence and could be formulated with several potential adjuvants of commercial relevance.

Antibody responses raised by different antigen–adjuvant combinations can exhibit variable binding and efficacy against naturally occurring variants of SARS-CoV-2 (37). We tested the binding of antibodies from the first study raised against RBD-Wuhan-Hu-1 and RBD-L452K-F490W to RBD molecules with mutations found in two recently reported SARS-CoV-2 variants of concern, 501Y.V1 and 501Y.V2, which were originally isolated in the United Kingdom and South Africa, respectively (Fig. 2*E*) (38). Antibodies from mice immunized with RBD-L452K-F490W with alum or SMNP retained binding to both RBD variants. Interestingly, antibodies raised with CpG adjuvant did not retain binding. These results suggest that immune responses elicited by RBD-L452K-F490W may protect against SARS-CoV-2 variants with the N501Y spike protein mutation.

**Fig. 3.**
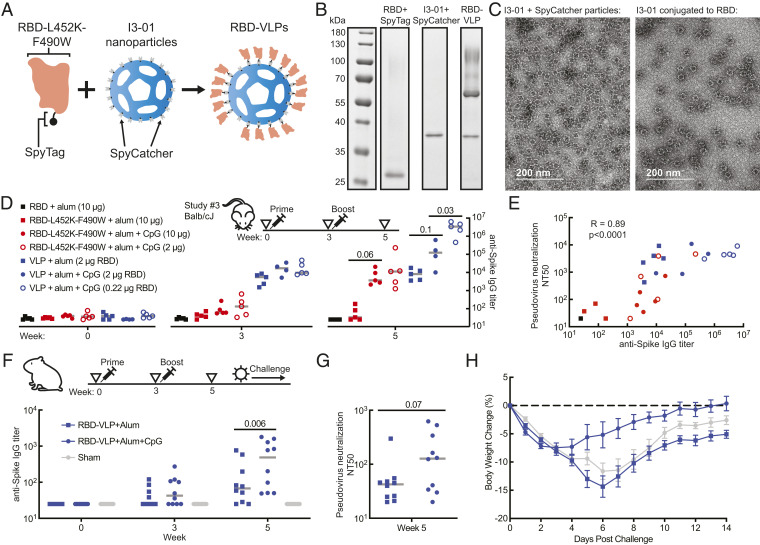
Immunogenicity and antigenicity of engineered RBD nanoparticles in mice and hamsters. (*A*) Schematic of nanoparticle assembly using SpyTag and SpyCatcher. (*B*) Reduced SDS-PAGE of nanoparticle components. (*C*) Negative stain electron microscopy of SpyCatcher-12GS-I3-01 nanoparticles before (*Left*) and after (*Right*) conjugation to RBD-L452K-F490W-GGDGGDGGDGG-SpyTag. (*D*) Titer of spike protein–specific IgG in study 3 mouse sera by ELISA. Data points represent individual animals. (*E*) Spearman correlation of anti-S protein IgG ELISA and pseudovirus neutralization from week 5 mouse sera. (*F*) Titer of spike protein–specific IgG in hamster sera by ELISA. (*G*) Titer of neutralizing antibody in week 5 hamster sera by SARS-CoV-2 pseudovirus neutralization assay. (*H*) Mean percent body weight change of hamsters in each group after challenge with SARS-CoV-2. Error bars represent SEs. Gray bars represent median values. Significance was determined by *t* test. *P* values are indicated on plots.

Multimeric display of subunit antigens like RBD on nanoparticle-based scaffolds provides a promising approach to enhance immunogenicity further and to reduce the amount of protein required for individual doses of a vaccine or the number of doses required (39, 40). Both attributes could facilitate broader global coverage for COVID-19 vaccines. We further modified the engineered RBD-L452K-F490W to include a peptide motif for covalently linking the antigen to a virus-like particle (VLP) via a transpeptidation reaction and produced the antigen similarly to the unmodified version (Fig. 3 *A* and *B* and *SI Appendix*, Fig. S3*A*) (41, 42). We conjugated the engineered antigen onto a designed self-assembling nanoparticle (i3-01) produced in bacteria (43). The resulting particles had ∼85% occupancy of the 60 available sites for antigenic display on each VLP (Fig. 3 *A* and *B* and *SI Appendix*, Fig. S3*C*). We confirmed that VLPs were correctly assembled by electron microscopy and size exclusion chromatography before and after conjugation (Fig. 3*C* and *SI Appendix*, Fig. S3*D*). In a third mouse study, we immunized Balb/cJ mice with 10 µg or 2 µg of RBD-L452K-F490W monomer, or doses of RBD-VLP constructs containing 2 µg or 0.22 µg of the RBD antigen, and boosted mice 21 d later (Fig. 3 *D* and *E*). We observed high antibody titers with a combination of alum and CpG1018 adjuvants for both the engineered RBD monomer and the RBD-VLP. We also evaluated pseudovirus neutralizing antibody titers, and observed that they correlated overall with anti-spike protein antibody titers (Fig. 3*E* and *SI Appendix*, Fig. S3*D*). Interestingly, we observed an enhanced anti-spike antibody response with a reduced dose of the RBD-VLP, but this effect was not significant for pseudovirus neutralization. To further assess the potential of the RBD-VLP to elicit an immune response with a low dose, we performed a fourth mouse study. We immunized mice with RBD-VLP doses containing 5 µg of RBD down to 0.06 µg, with alum and CpG1018, and boosted after 21 d. All doses induced seroconversion with strong titers of neutralizing antibodies (*SI Appendix*, Fig. S3*E*). These results suggest that multimeric display of the RBD may enable a low-dose formulation. Since dose size could have large implications for manufacturing and global access, formulation of RBD-VLPs with small doses warrants further investigation.

To evaluate whether the RBD-L452K-F490W antigen could protect against live SARS-CoV-2 virus, we immunized golden Syrian hamsters with the RBD-VLP containing 2 µg of RBD antigen formulated with either alum or alum and CpG1018—a commercial good manufacturing practice–grade adjuvant—with a prime and a boost after 3 wk. We observed anti-spike antibodies and pseudovirus neutralizing antibodies in animals that received both formulations, and a significantly higher response from the alum + CpG1018 formulation (Fig. 3 *F* and *G*). Following the boost, we challenged the hamsters with SARS-CoV-2 and monitored for body weight change and viral titer postchallenge. Animals that received the RBD-VLP with alum + CpG recovered in weight faster than the control group (*P* = 0.04, day 6 postchallenge) (Fig. 3*H* and *SI Appendix*, Fig. S3*F*). Interestingly, the formulation with alum did not result in a significantly different weight change than placebo. Across all vaccinated animals, absolute body weight change was positively correlated with the measured titer of neutralization antibody from sera—that is, animals with higher antibody titers tended to lose less weight (*SI Appendix*, Fig. S3*G*). These studies demonstrate one potential presentation of the RBD-L452K-F490W as a vaccine antigen on a nanoparticle and its efficacy reducing the effects of SARS-CoV-2 in the hamster model.

## Discussion

Here, we have demonstrated an engineered variant of SARS-CoV-2 RBD that exhibits improved biomolecular attributes that make it well suited for further development for large-volume manufacturing of low-cost vaccine candidates. This design also shows improved immunogenicity and a more durable immune response in mice compared to the original Wuhan-Hu-1 sequence for RBD used in current vaccines when formulated with multiple commercially relevant single adjuvants. We hypothesize that the apparent increase in immunogenicity is due to the enhanced stability of the molecule. These promising results motivate further studies to assess the potential of engineered variants like this one to improve immunogenicity and potency of RBD-based designs in nonhuman primates and ultimately clinical studies.

Improving the designs of vaccines for COVID-19 will remain critical as new variants like 501Y.V1/V2 continue to emerge with mutations in the RBD. Such adaptations by the virus could reduce the effectiveness of interventions like monoclonal antibodies and current vaccines based on the original Wuhan-Hu-1 strain (44, 45). Antibodies raised against the engineered RBD reported here exhibit heterotypic binding to both 501Y.V1 and V2. Interestingly, these viral variants also exhibit enhanced binding to ACE2, similar to the design here (46), and genomic sequences for reported strains containing mutations at E484 also show co-occurrences with changes at F490 (47). The ACE2 RBM remains a critical epitope for neutralization of emerging variants (48). Other recent variants like B.1.429 (epsilon) and B.1.617 (delta) that also show escape from known neutralizing antibodies for SARS-CoV-2 contain a strikingly similar change in the first residue identified in our engineered design (L452R) (49, 50).

The RBD remains an important candidate for primary vaccines and boosters due to the high concentration of neutralizing epitopes and CD4^+^ T cell epitopes (48, 51). (We note that other domains of the spike protein like the N-terminal domain, however, can also elicit neutralizing antibodies) (52). The modifications to RBD we demonstrated here are limited to the ACE2 RBM, and, therefore, are compatible in principle with any vaccine based on presentation of the S protein, the S1 subunit, or the RBD. Despite containing many neutralizing epitopes, currently approved protein vaccines based on the S protein of the Wuhan-Hu-1 strain have shown reduced neutralization of the prevailing variants like 501Y.V2 (53). Interestingly, the RBD has been shown to effectively boost immune responses against emerging variants (54), further motivating vaccine candidates for both SARS-CoV-2 and betacoronaviruses, more generally, in multimeric displays like nanoparticles (55). We demonstrated here that the engineered RBD can be protective when formulated on nanoparticles and could be combined with other specific mutations identified from naturally occurring variants. The increased manufacturability and immunogenicity of the design presented here may afford further insights to improve the breadth of protection afforded by SARS-CoV-2 vaccine candidates and ultimately affordable and accessible vaccines.

## Materials and Methods

### Strains.

All strains were derived from wild-type *K. phaffii* (NRRL Y-11430), in a modified base strain (RCR2_D196E, RVB1_K8E) described previously (56). Genes containing RBD variants were codon optimized, synthesized (Integrated DNA Technologies), and cloned into a custom vector. *K. phaffii* strains were transformed as described previously (57).

### Cultivations.

Strains for initial characterization and titer measurement were grown in 3 mL of culture in 24-well deep-well plates (25 °C, 600 rpm), and strains for protein purification were grown in 200 mL of culture in 1-L shake flasks (25 °C, 250 rpm). Cells were cultivated in complex media (potassium phosphate buffer pH 6.5, 1.34% nitrogen base w/o amino acids, 1% yeast extract, 2% peptone). Cells were inoculated at 0.1 OD_600_, outgrown for 24 h with 4% glycerol feed, pelleted, and resuspended in fresh media with 3% methanol to induce recombinant gene expression. Supernatant samples were collected after 24 h of production, filtered, and analyzed. InSCyT bioreactors were operated as described previously (21).

### Transcriptome Analysis.

Cell were harvested after 18 h of production at 3-mL plate scale. RNA was extracted and purified according to the Qiagen RNeasy kit (Cat. No. 74104), and RNA quality was analyzed to ensure RNA quality number >6.5. RNA libraries were prepared using the 3′DGE method and sequenced on an Illumina Nextseq to generate paired reads of 20 (read 1) and 72 bp (read 2). Sequenced mRNA transcripts were demultiplexed using sample barcodes and PCR duplicates were removed by selecting one sequence read per unique molecular identifier (UMI) using a custom python script. Transcripts were quantified with Salmon version 1.1.0 (58) and selective alignment using a target consisting of the *K. phaffii* transcripts, the RBD-N1del, P[8], and selectable marker transgene sequences and the *K. Phaffii* genome as a selective alignment decoy. Expression values were summarized with tximport version 1.12.3 (59) and edgeR version 3.26.8 (60, 61). Expression was visualized using log_2_(counts per million + 1) values. Gene set enrichment analysis (GSEA) was performed with GSEA 4.1.0 using Wald statistics calculated by DESeq2 (62) and gene sets from yeast GO Slim (63). Raw data used in this study can be obtained from the NCBI Gene Expression Omnibus (accession no. GSE172054).

### Protein Purification.

Protein purification for nonclinical studies and end-to-end manufacturing was carried out on the purification module of the InSCyT system as described previously (21). All columns were equilibrated in the appropriate buffer prior to each run. Product-containing supernatant was adjusted to pH 4.5 using 100 mM citric acid. The adjusted supernatant was loaded into a prepacked CMM HyperCel column (5 mL) (Pall Corporation), reequilibrated with 20 mM sodium citrate pH 5.0, washed with 20 mM sodium phosphate pH 5.8, and eluted with 20 mM sodium phosphate pH 8.0, 150 mM NaCl. Eluate from column 1 above 15 mAU was flowed through a 1-mL prepacked HyperCel STAR AX column (Pall Corporation). Flow-through from column 2 above 15 mAU was collected.

### Analytical Assays for Protein Characterization.

Purified protein concentrations were determined by absorbance at A280 nm. SDS-PAGE was carried out as described previously (21). Supernatant titers were measured by reverse phase liquid chromatography, and normalized by cell density, measured by OD_600_.

### Biolayer Interferometry.

Biolayer interferometry was performed using the Octet Red96 with Protein A (ProA) biosensors (Sartorius ForteBio), which were hydrated for 15 min in kinetics buffer prior to each run. Kinetics buffer comprising 1× phosphate-buffered saline (PBS) pH 7.2, 0.5% bovine serum albumin (BSA), and 0.05% Tween-20 was used for all dilutions, baseline, and disassociation steps. CR3022 and ACE2-Fc were used in the assay at concentrations of 2 and 10 µg/mL, respectively. Samples were loaded in a 96-well black microplate (Greiner Bio-One) at starting concentrations of 15 and 10 µg/mL, respectively. Seven 1:1 serial dilutions and a reference well of kinetics buffer were analyzed for each sample. Association and dissociation were measured at 1,000 rpm for 300 and 600 s, respectively. Binding affinity was calculated using the Octet Data Analysis software v10.0 (Pall ForteBio), using reference subtraction, baseline alignment, interstep correction, Savitzky-Golay filtering, and a global 1:1 binding model.

### Size Exclusion Chromatography.

Size exclusion high-performance liquid chromatography (HPLC) analysis was performed on an Agilent 1260 HPLC system controlled using OpenLab CDS software (Agilent Technologies). The analysis was performed using an AdvanceBio SEC column (Agilent Technologies, 4.6 × 300 mm, 300 Å, 2.7 µm) and AdvanceBio SEC guard column (Agilent Technologies, 4.6 × 50 mm, 300 Å, 2.7 µm). The column was operated at a flow rate of 0.25 mL/min and ambient temperature. The mobile phase buffer was 150 mM sodium phosphate (Sigma-Aldrich), pH 7.0. Total method run time was 30 min and sample injection volumes were 10 µL. A diode array detector was set for absorbance detection at 214 nm. Data analysis was completed using Agilent’s OpenLab CDS Data Analysis.

### Reverse Phase Chromatography.

Reverse phase HPLC analysis was performed on an Agilent 1260 HPLC system controlled using OpenLab CDS software (Agilent Technologies). Antigen concentration was determined using a PLRP-S column (2.1 × 150 mm, 300 Å, 3 µm) operated at 0.6 mL/min and 80 °C (Agilent Technologies) on an HPLC equipped with a diode array detector set for absorbance detection at 214 nm and buffer A was 0.1% (vol/vol) trifluoroacetic acid (TFA) in water and buffer B was 0.1% (vol/vol) TFA, 0.5% (vol/vol) water in acetonitrile. A gradient of 39 to 43% B was performed over 9 min; total method run time was 18 min. Sample injection volumes were 50 µL. A diode array detector was set for absorbance detection at 214 nm. Data analysis was completed using OpenLab CDS Data Analysis (Agilent Technologies).

### Mass Spectrometry.

Intact mass analysis was performed on a 6530B quadrupole time-of-flight liquid chromatograph mass spectrometer (LC-MS) with a 1290 series HPLC (Agilent Technologies). Mobile phase A consisted of LC-MS grade water with 0.1% formic acid, and mobile phase B was LC-MS grade acetonitrile with 0.1% formic acid. About 1.0 µg of protein for each sample was injected, bound to a PLRP-S column (2.1 mm × 50 mm, 5 μm, 300 Å) (Agilent Technologies), desalted, and subjected to electrospray ionization. The LC gradient comprised 5 to 95% B over 30 min at a flow rate of 0.4 mL/min. A blank injection between each sample was performed as a wash step, consisting of an LC gradient of 5 to 95% B over 6 min. The electrospray ionization parameters were as follows: 325 °C drying gas temperature, 10 L/min drying gas flow, 30 psig nebulizer, 4,000 V Vcap, and 325 V fragmentor voltage. Mass spectra were collected from 500 to 3,200 *m/z* at a scan rate of 1 spectra/s. MS spectra were processed using MassHunter Bioconfirm software (v B.10.0, Agilent Technologies) with a deconvolution range of 10 to 50 kDa, using a mass step of 1 Da.

### Far-Ultraviolet Circular Dichroism.

Circular dichroism (CD) spectroscopy was performed using a Chirascan-plus CD spectrometer (Applied Photophysics Ltd.) equipped with a six-cuvette position Peltier temperature controller (Quantum Northwest) and a high-performance solid-state detector. The lamp (150 W air-cooled Xe arc) housing, monochromator, and sample compartment were continuously purged with N_2_ gas. The 10 °C CD spectra of RBD samples at 0.2 mg/mL were collected in triplicate in the range of 280 to 200 nm using quartz cuvettes (1-mm path length) sealed with a Teflon stopper (Starna Cells Inc.). Data were subjected to a three-point Savitzky-Golay smoothing filter using Chirascan software (Applied Photophysics) and the ellipticity of the buffer was subtracted from all sample measurements.

### Static Light Scattering vs. Temperature.

Static light scattering measurements as a function of temperature were made in triplicate using a dual emission PTI QM-40 Spectrofluorometer (Horiba Scientific) equipped with a four-position cell holder Peltier temperature control device, a high-power continuous 75 W short-arc Xe lamp (Ushio), and a Hamamatsu R1527 photomultiplier tube. Data were collected using FelixGX software (Horiba Scientific) in 10-mm path length quartz cuvettes. RBD samples at 0.2 mg/mL were examined as a function of temperature (10 °C to 90 °C) using an excitation wavelength of 295 nm. Static light scattering signal at 295 nm was collected at a 1.25 °C interval with a 2-min equilibration at each temperature. The light scattering signal of the buffer was subtracted from all sample measurements and the light scattering intensity at 295 nm was plotted as a function of temperature.

### Differential Scanning Calorimetry.

Differential scanning calorimetry (DSC) was performed in triplicate using an auto-VP capillary differential scanning calorimeter (MicroCal/GE Health Sciences) equipped with Tantalum sample and reference cells were pressurized at ∼60 psi with nitrogen. RBD samples at 0.2 mg/mL were loaded in the autosampler tray held at 4 °C and scans were completed from 10 °C to 90 °C using a scan rate of 60 °C/h and a prescan thermostat of 15 min. Buffer subtraction and concentration normalization were performed using Origin (OriginLab). Data analysis was performed using the MicroCal LLC DSC plug-in for the Origin 7.0 software package.

### Mouse Study 1: Soluble RBD Studies.

The immunogenicity of RBD-L452K-F490W compared to RBD was evaluated in vivo in mice. All procedures were approved by the Massachusetts Institute of Technology Institutional Animal Care and Use Committee following local, state, and federal regulations. Immunization studies were carried out using age-matched 6- to 8-wk-old Balb/cJ female mice purchased from The Jackson Laboratory (strain 000651). Mice were immunized on day 0 and day 21 with 5 µg RBD plus adjuvant: 50 µg alum Alhydrogel (Invivogen), 30 µg CpG1826 (Invivogen), or 5 µg saponin monophosphoryl lipid A nanoparticles (SMNP). SMNP was synthesized in-house, where dose is reported as the amount of saponin administered. Immunizations were administered via subcutaneous injection in 100 µL PBS at the tail base (2 × 50 µL bilateral injections, one on each side of the tail base). Blood was collected by cheek or retroorbital bleed for enzyme-linked immunosorbent assay (ELISA) antibody analysis on weeks 2, 3, 4, and then every 2 wk thereafter. Serum was isolated from blood using serum separator tubes, centrifuged at 10,000 × *g* for 5 min at 4 °C, then stored at −80 °C.

### RBD-Specific ELISAs.

Anti-RBD IgG was measured in mouse serum by ELISA. To capture serum antibodies from immunized mice, Costar polystyrene high binding 96-well plates (Corning) were coated directly with RBD antigen at 2 µg/mL in PBS overnight at 4 °C, then blocked with PBS + 2% BSA for 2 h at 25 °C. Mouse sera were diluted in block buffer (PBS + 2% BSA) starting at 1:100 or 1:200 followed by 4× serial dilutions and incubated in plates for 2 h at 25 °C, followed by detection with 1:5,000 goat anti-mouse IgG–horseradish peroxidase (HRP) (Bio-Rad) in block buffer for 1 h. Plates were developed using tetramethylbenzidine (TMB) substrate for 1 to 20 min and stopped with 2N sulfuric acid. For all titer analyses, samples directly compared across groups were developed for the same amount of time. Cutoff titers are reported as inverse dilutions giving a 0.2 HRP absorbance (A450 − A540).

### Mouse Study 2: RBD Adjuvant Studies.

The immunogenicity of RBD-L452K-F490W in combination with various adjuvants was evaluated in 7- to 8-wk-old C57BL/6J female mice (Charles River, strain 000634) at the Vaccine Formulation Institute (VFI, Switzerland). All animal work was performed in accordance with the Swiss Federal Animal Protection Act. Mice were immunized via intramuscular injection with RBD antigen and adjuvants including aluminum hydroxide (AlOH), SWE (64) (squalene-in-water emulsion), SQ (SWE + QS21 saponin, VFI), SMQ (65) (squalene-in-water emulsion + synthetic TLR4L and QS21, VFI), LQ (65) (neutral liposomes + QS21, VFI), or LMQ (65) (neutral liposome + synthetic TLR4L and QS21 saponin, VFI). The TLR4 agonist was used at a final concentration of 2 µg/dose and the QS21 saponin at 5 µg/dose. The spike prefusion trimer (produced previously, ref. 39) adjuvanted with SWE was used as a benchmark in this experiment as it has been shown to induce high titers of neutralizing antibodies. All formulations were fully characterized for adjuvant physicochemical properties and antigen integrity. Blood samples were collected on day 42 and sera tested for RBD-specific antibodies in ELISA with the following modifications: Plates were coated with 1.25 µg/mL soluble RBD (produced previously) (39) and mouse serum Ig was detected using a goat anti-mouse Ig coupled to HRP (Southern Biotech) diluted at 1/6,000.

### Production of RBD Nanoparticles.

Plasmids encoding the SpyCatcher-I3-01 nanoparticles were transformed into Lemo21 (DE3) *Escherichia coli* cells grown in lysogeny broth (LB) media supplemented with 50 mg/L kanamycin. Expression cultures were induced with 1 mM isopropyl β-D-1-thiogalactopyranoside (IPTG) and shaken at 220 rpm at 18 °C for 18 h. Cell cultures were harvested by centrifugation at 4,000 × *g* for 15 min. Media were decanted and the cell pellets resuspended in 50 mM Tris pH 8.0, 150 mM NaCl, 0.05 mg/mL DNase, 0.05 mg/mL RNase, 1 mM phenylmethylsulfonyl fluoride (PMSF), and lysed using a microfluidizer. Lysates were clarified by centrifugation at 24,500 × *g* for 30 min and the insoluble and soluble fractions analyzed by SDS-PAGE. Ammonium sulfate was added to 20% saturation to the supernatant and incubated with gentle rotation at room temperature for 1 h. Samples were clarified by centrifugation at 14,000 rpm for 30 min and solubility was analyzed by SDS-PAGE. The insoluble pellet containing the protein of interest was resuspended in 50 mM Tris pH 8.0, 150 mM NaCl, and placed in a rock bath at 80 °C for 20 min. Insoluble and soluble fractions were again separated by centrifugation as in the previous step. Supernatants containing the protein of interest were concentrated using centrifugal filter units (Merck Millipore). The nanoparticles were further purified by SEC using a Superose 6 Increase 10/300 GL column (Cytiva) preequilibrated in 50 mM Tris pH 8.0, 150 mM NaCl. Fractions containing the pure nanoparticle were pooled and filtered using a 0.22-μm filter for further analysis. The nanoparticles were quantified by ultraviolet-visible spectrophotometry (UV-Vis) using an Agilent Technologies Cary 8454.

Endotoxin was removed using Pierce High Capacity Endotoxin Removal Spin Columns (Thermo Scientific) to below 10 EU/mL and quantified using a Pierce Chromogenic Endotoxin Quant Kit (Thermo Scientific). I3-01-spycatcher and RBD-spytag were conjugated by incubation overnight at 4 °C in 20 mM sodium phosphate, 150 mM NaCl, pH 8 buffer, with a 1:1.5 I3-01-spycatcher:RBD-spytag molar ratio. Excess RBD was removed with a 100-kDa molecular weight cutoff Amicon Ultra-4 centrifugal filter (Millipore). RBD valency was determined using SDS-PAGE densitometry in ImageJ.

### Negative-Stain Electron Microscopy.

Solution with conjugated RBD-VLP nanoparticles (7 µL) was incubated on a 200-mesh copper grid coated with a continuous carbon film for 60 s. Excess liquid was removed, and the film was incubated in 10 µL of 2% uranyl acetate. The grid was dried at room temperature and mounted on a JEOL single tilt holder in the TEM column. The specimen was cooled by liquid nitrogen and imaged on a JEOL 2100 FEG microscope with a minimum dose to avoid sample damage. The microscope was operated at 200 kV with magnification at 10,000 to 60,000×. Images were recorded on a Gatan 2Kx2K UltraScan CCD camera.

### Mouse Studies 3 and 4: RBD-VLP Studies.

Balb/cJ female mice, ages 6 to 8 wk were purchased from The Jackson Laboratory (strain 000651). Mice were immunized on day 0 and day 21 with the indicated doses of RBD-VLPs or soluble RBD-L425K-F490W monomer as a control; all groups were adjuvanted with 40 µg alum + 10 µg CpG. Immunizations were administered via bilateral intramuscular injection. Peripheral blood was collected via the submandibular route at days 0, 21, and 35, and serum was isolated for immunologic assays. Studies were conducted in compliance with all relevant local, state, and federal regulations and approved by the Beth Israel Deaconess Medical Center Institutional Animal Care and Use Committee.

### Spike Protein-Specific ELISAs.

Nunc Immuno MaxiSorp 96-well plates (Thermo Scientific) were coated with SARS-CoV-2 spike protein (Sino Biological) at 1 µg/mL in PBS and incubated overnight at 4 °C. After incubation, plates were washed once with wash buffer (0.05% Tween-20 in 1× PBS), then blocked with casein for 2 to 3 h at 25 °C. Mouse or hamster sera were diluted in casein block buffer starting at 1:25 followed by 3× serial dilutions and incubated in plates for 1 h at 25 °C. Plates were then washed three times and incubated with either rabbit anti-mouse IgG-HRP (Jackson Immuno) or goat anti-hamster IgG(H+L)-HRP (SouthernBiotech) diluted in casein for mouse or hamster samples, respectively. After 1-h incubation at 25 °C, plates were washed three times, then developed using TMB substrate (SeraCare). Development was halted using stop solution (SeraCare). For each sample, ELISA endpoint titer was calculated in GraphPad Prism software, using a four-parameter logistic curve fit to calculate the reciprocal serum dilution that yields an absorbance value (450 nm) of 0.2.

### Pseudovirus Neutralization Assays.

The SARS-CoV-2 pseudoviruses expressing a luciferase reporter gene were generated in an approach similar to previous descriptions (66–68). Briefly, the packaging plasmid psPAX2 (AIDS Resource and Reagent Program), luciferase reporter plasmid pLenti-CMV Puro-Luc (Addgene), and spike protein expressing pcDNA3.1-SARS CoV-2 SΔCT were cotransfected into HEK293T cells by Lipofectamine 2000 (Thermo Fisher). The supernatants containing the pseudotype viruses were collected 48 h posttransfection, which were purified by centrifugation and filtration with a 0.45-µm filter. To determine the neutralization activity of the plasma or serum samples from animals, HEK293T-hACE2 cells were seeded in 96-well tissue culture plates at a density of 1.75 × 10^4^ cells/well overnight. Threefold serial dilutions of heat-inactivated plasma samples were prepared and mixed with 50 µL of pseudovirus. The mixture was incubated at 37 °C for 1 h before adding to HEK293T-hACE2 cells. Forty-eight hours after infection, cells were lysed in Steady-Glo Luciferase Assay (Promega) according to the manufacturer’s instructions. SARS-CoV-2 neutralization titers were defined as the sample dilution, at which a 50% reduction in relative light unit (RLU) was observed relative to the average of the virus control wells.

### Immunization of Hamsters.

Male and female Syrian golden hamsters (Envigo), 7 to 8 wk of age, were randomly distributed into three groups. Hamsters were immunized via intramuscular injection with a prime at week 0, followed by a boost at week 3 of 1) RBD-J-ST-i3 VLPs containing 2 µg of RBD protein + 40 µg of alum, 2) RBD-J-ST-i3 VLPs containing 2 µg of RBD protein + 40 µg of alum + 100 µg of CpG, or 3) sham. Peripheral blood was drawn via the retroorbital route at baseline (week 0), week 3, and week 5 to collect serum for immunologic assays. At week 5, hamsters were challenged with 1.99 × 10^4^ TCID50 SARS-CoV-2, derived from USA-WA1/2020 (NR-53780, BEI Resources). Challenge virus was administered in 100 µL of total volume by the intranasal route (50 µL in each nare). After challenge, body weights of hamsters were monitored daily. Studies were conducted in compliance with all relevant local, state, and federal regulations and approved by the Bioqual Institutional Animal Care and Use Committee.

## Supplementary Material

Supplementary File

## Data Availability

Gene expression data have been deposited in GEO Omnibus (GSE172054).
