# Supplementary material for: Engineered SARS-CoV-2 receptor binding domain improves manufacturability in yeast and immunogenicity in mice
Source: Proc Natl Acad Sci U S A. 2021 Sep 7;118(38):e2106845118. doi: 10.1073/pnas.2106845118 (PMC8463846; doi:10.1073/pnas.2106845118)
Supplement: Supplementary File [file pnas.2106845118.sapp.pdf]

Engineered SARS-CoV-2 receptor binding domain improves manufacturability in yeast and immunogenicity in mice

Supporting information

Neil C. Dalvie[1,2]^, Sergio A. Rodriguez-Aponte[2,3]^, Brittany L. Hartwell[2,4], Lisa H. Tostanoski[5], Andrew M. Biedermann[1,2], Laura E. Crowell[1,2], Kawaljit Kaur[6], Ozan S. Kumru[6], Lauren Carter[7,8], Jingyou Yu[5], Aiquan Chang[5,9], Katherine McMahan[5], Thomas Courant[10], Celia Lebas[10], Ashley A. Lemnios[2], Kristen A. Rodrigues[2,4,11], Murillo Silva[2], Ryan S. Johnston[2], Christopher A. Naranjo[2], Mary Kate Tracey[2], Joseph R. Brady[1,2], Charles A. Whittaker[2], Dongsoo Yun[2], Natalie Brunette[7,8], Jing Yang Wang[7,8], Carl Walkey[7,8], Brooke Fiala[7,8], Swagata Kar[12], Maciel Porto[12], Megan Lok[12], Hanne Andersen[12], Mark G. Lewis[12], Kerry R. Love[1,2], Danielle L. Camp[2], Judith Maxwell Silverman[13], Harry Kleanthous[14], Sangeeta B. Joshi[6], David B. Volkin[6], Patrice M. Dubois[10], Nicolas Collin[10], Neil P. King[7,8], Dan H. Barouch[4,5,9,15], Darrell J. Irvine[2,3,4,16], J. Christopher Love[1,2]

[1]Department of Chemical Engineering, Massachusetts Institute of Technology, Cambridge, Massachusetts 02139, USA
[2]The Koch Institute for Integrative Cancer Research, Massachusetts Institute of Technology, Cambridge, Massachusetts 02139, USA
[3]Department of Biological Engineering, Massachusetts Institute of Technology, Cambridge, Massachusetts 02139, USA
[4]Ragon Institute of MGH, MIT, Harvard, Cambridge, MA 02139, USA
[5]Center for Virology and Vaccine Research, Beth Israel Deaconess Medical Center, Harvard Medical School, Boston, MA, USA
[6]Department of Pharmaceutical Chemistry, Vaccine Analytics and Formulation Center, University of Kansas, Lawrence, Kansas, 66047, United States
[7]Department of Biochemistry, University of Washington, Seattle, WA 98195, USA
[8]Institute for Protein Design, University of Washington, Seattle, WA 98195, USA
[9]Harvard Medical School, Boston, MA 02115, USA
[10]Vaccine Formulation Institute, 1228 Plan-Les-Ouates, Geneva, Switzerland
[11]Harvard-MIT Health Sciences and Technology, Institute for Medical Engineering and Science, Massachusetts Institute of Technology, Cambridge, MA 02139, USA
[12]Bioqual, Inc., Rockville, MD 20850, USA
[13]Bill & Melinda Gates Medical Research Institute, Cambridge, MA 02139, USA
[14]Bill&Melinda Gates Foundation, Seattle, WA 98109, USA
[15]Massachusetts Consortium on Pathogen Readiness, Boston, MA 02115, USA
[16]Howard Hughes Medical Institute, Chevy Chase, MD 20815, USA

^Contributed equally

*Correspondence to: clove@mit.edu

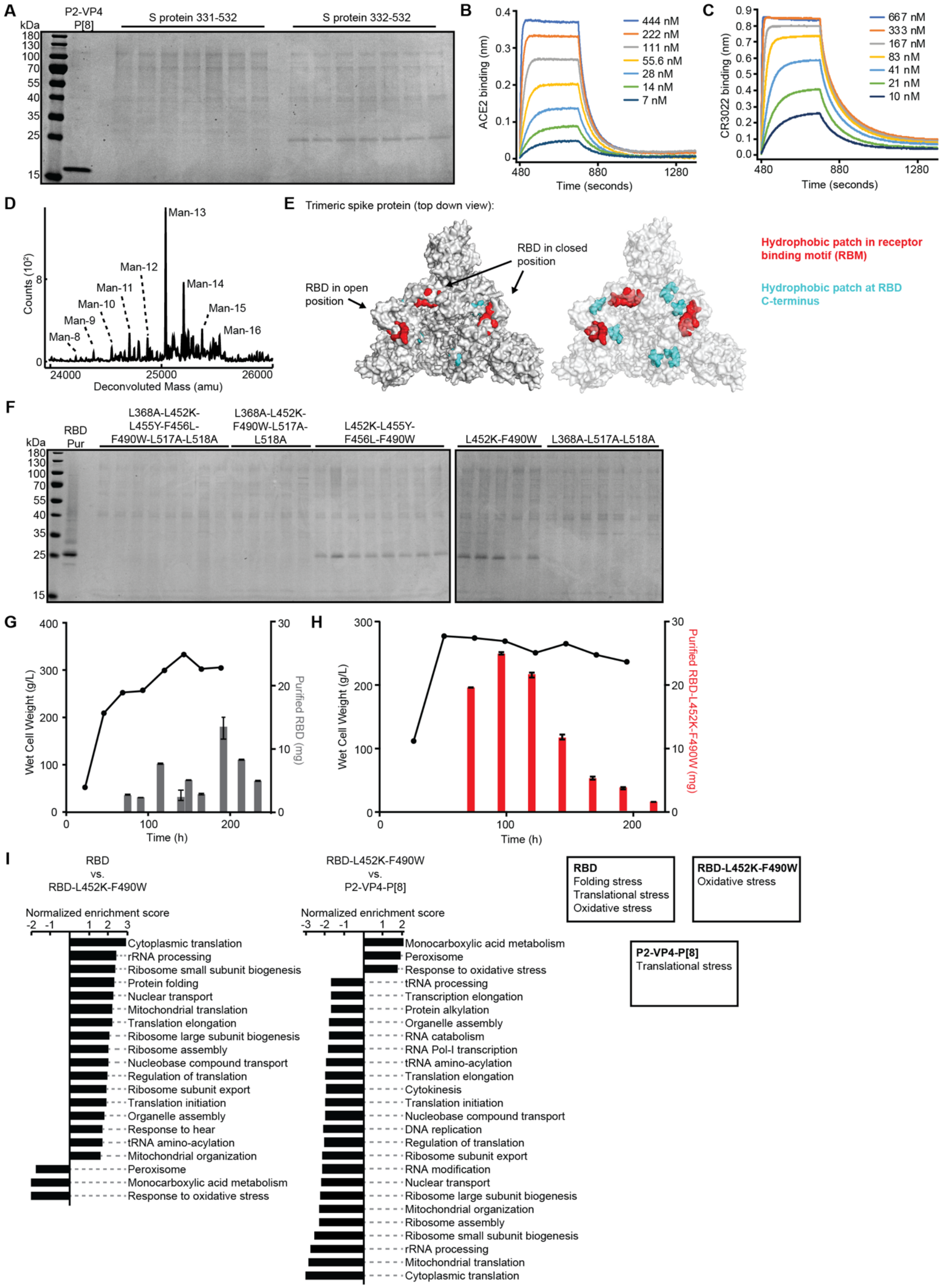

A
kDa
P2-VP4 P[8]
S protein 331-532
S protein 332-532
180
130
100
70
55
40
35
25
15
B
ACE2 binding (nm)
Time (seconds)
444 nM
222 nM
111 nM
55.6 nM
28 nM
14 nM
7 nM
C
CR3022 binding (nm)
Time (seconds)
667 nM
333 nM
167 nM
83 nM
41 nM
21 nM
10 nM
D
Counts (10²)
Deconvoluted Mass (amu)
Man-8
Man-9
Man-10
Man-11
Man-12
Man-13
Man-14
Man-15
Man-16
E
Trimeric spike protein (top down view):
RBD in open position
RBD in closed position
Hydrophobic patch in receptor binding motif (RBM)
Hydrophobic patch at RBD C-terminus
F
kDa
RBD Pur
L368A-L452K-L455Y-F456L-F490W-L517A-L518A
L368A-L452K-F490W-L517A-L518A
L452K-L455Y-F456L-F490W
L452K-F490W
L368A-L517A-L518A
G
Wet Cell Weight (g/L)
Purified RBD (mg)
Time (h)
H
Wet Cell Weight (g/L)
Purified RBD-L452K-F490W (mg)
Time (h)
I
RBD vs. RBD-L452K-F490W
Normalized enrichment score
Cytoplasmic translation
rRNA processing
Ribosome small subunit biogenesis
Protein folding
Nuclear transport
Mitochondrial translation
Translation elongation
Ribosome large subunit biogenesis
Ribosome assembly
Nucleobase compound transport
Regulation of translation
Ribosome subunit export
Translation initiation
Organelle assembly
Response to hear
tRNA amino-acylation
Mitochondrial organization
Peroxisome
Monocarboxylic acid metabolism
Response to oxidative stress
RBD-L452K-F490W vs. P2-VP4-P[8]
Normalized enrichment score
Monocarboxylic acid metabolism
Peroxisome
Response to oxidative stress
tRNA processing
Transcription elongation
Protein alkylation
Organelle assembly
RNA catabolism
RNA Pol-I transcription
tRNA amino-acylation
Translation elongation
Cytokinesis
Translation initiation
Nucleobase compound transport
DNA replication
Regulation of translation
Ribosome subunit export
RNA modification
Nuclear transport
Ribosome large subunit biogenesis
Mitochondrial organization
Ribosome assembly
Ribosome small subunit biogenesis
rRNA processing
Mitochondrial translation
Cytoplasmic translation
RBD
Folding stress
Translational stress
Oxidative stress
RBD-L452K-F490W
Oxidative stress
P2-VP4-P[8]
Translational stress

Fig. S1

Initial expression and manufacturing of RBD variants

(A) Reduced SDS-PAGE of cultivation supernatants of initial RBD variants. Each lane represents a unique clone after transformation. (B,C) Binding of RBD to human ACE2-IgG fusion protein (B) and CR3022 neutralizing antibody (C) by biolayer interferometry. (D) Mass spectrum of purified RBD with labeled specific glycan peaks. Man = mannose. (E) Structural rendering of the spike protein trimer, from top down view Two predicted hydrophobic patches that exist on the surface of the RBD are labeled. Rendering on the right is transparent to reveal the second patch. (F) Reduced SDS-PAGE of cultivation supernatants of RBD variants. (F-G) Yields for production of RBD (G) and RBD-L452K-F490W (H). Wet cell weight and purified pools of RBD variants are shown. (I) Gene set enrichment analysis comparing strains expressing RBD, RBD-L452K-F490W, and a rotavirus VP8 fragment (left); summary of upregulated cellular processes in each strain (right).

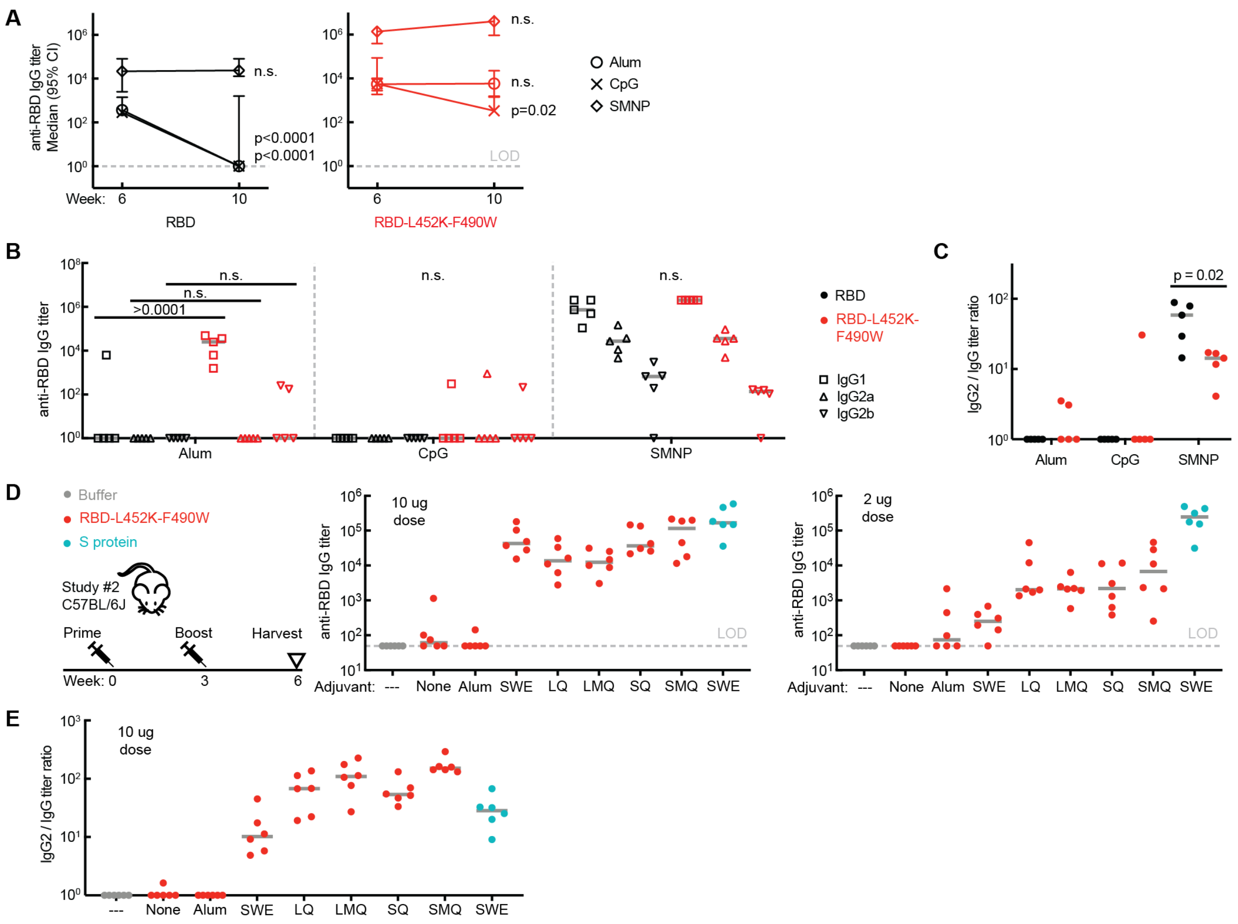

Fig. S2

Immunogenicity of engineered RBD formulated with single adjuvants

(A) Comparison of median values of anti-RBD IgG antibody titers from Study #1 mouse sera from weeks 6 and 10. Error bars represent 95% confidence intervals. (B) Titer of RBD-specific IgG1, IgG2a, and IgG2b antibodies in week 8 mouse sera by ELISA. Data points represent individual animals. (C) Log ratio of IgG2 (IgG2a+IgG2b) to total IgG (IgG1+IgG2a+IgG2b+IgG3) antibody titers. (D) Titer of RBD-specific total Ig in Study #2 mouse sera by ELISA. RBD-L452K-F490W was administered in 2 μg or 10 μg doses at weeks 0 and 3 with different adjuvants. (E) Log ratio of IgG2 (IgG2b+IgG2c) to total IgG antibody titers.

Data points represent individual animals. Gray lines represent median values. Significance was determined by t-test, with Holm-Sidak correction. LOD = limit of detection.

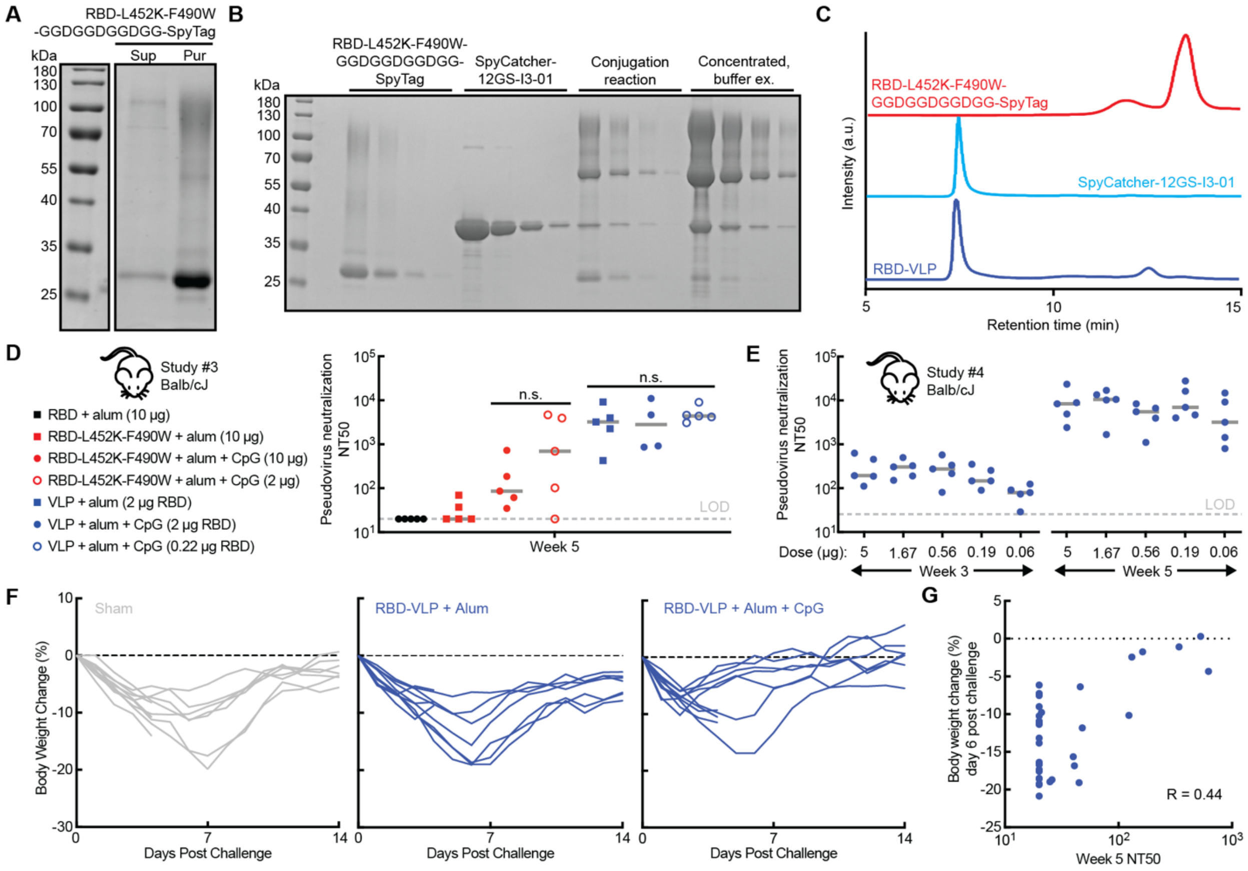

Figure S3

Production, characterization, and efficacy of RBD-VLP nanoparticles

(A) Reduced SDS-PAGE of RBD-L452K-F490W-GGDGGDGGDGG-SpyTag purification. Sup = cultivation supernatants, Pur = purified protein. (B) Reduced SDS-PAGE of purified RBD-L452K-F490W-GGDGGDGGDGG-SpyTag, SpyCatcher-12GS-I3-01 (produced in *E. coli*), nanoparticles after the conjugation reaction, and nanoparticles after buffer exchange and filter concentration. Gel replicates are serial 3x dilutions of each sample. (C) Size exclusion chromatography of nanoparticles before and after the conjugation reaction. (D) Titer of neutralizing antibody in Study #3 week 5 mouse sera from SARS-CoV-2 pseudovirus neutralization assay. (E) Titer of neutralizing antibody in Study #4 mouse sera from SARS-CoV-2 pseudovirus neutralization assay for the RBD-VLP dosing study. (F) Change in body weight of individual hamsters after challenge with SARS-CoV-2. Three animals in each group underwent

scheduled necropsies at day 4. (G) Spearman correlation of percentage weight change at day 6 post challenge with neutralizing antibody titer in sera sampled in week 5.

Data points represent individual animals. Gray lines represent median values. LOD = limit of detection.

Table S1 Animal studies in this manuscript

<table>
<tr><th></th><th>Antigen</th><th>Antigen dose</th><th>Adjuvant</th><th>N</th></tr>
<tr><td rowspan="6">Mouse study #1

Balb/cJ</td><td rowspan="3">RBD</td><td rowspan="3">5 µg</td><td>Alum</td><td>5</td></tr>
<tr><td>CpG</td><td>5</td></tr>
<tr><td>SMNP</td><td>5</td></tr>
<tr><td rowspan="3">RBD-L452K-F490W</td><td rowspan="3">5 µg</td><td>Alum</td><td>5</td></tr>
<tr><td>CpG</td><td>5</td></tr>
<tr><td>SMNP</td><td>5</td></tr>
<tr><td rowspan="17">Mouse study #2

C57BL/6J</td><td>Buffer</td><td></td><td>None</td><td>6</td></tr>
<tr><td rowspan="7">RBD-L452K-F490W</td><td rowspan="7">10 µg</td><td>None</td><td>6</td></tr>
<tr><td>Alum</td><td>6</td></tr>
<tr><td>SWE</td><td>6</td></tr>
<tr><td>LQ</td><td>6</td></tr>
<tr><td>LMQ</td><td>6</td></tr>
<tr><td>SQ</td><td>6</td></tr>
<tr><td>SMQ</td><td>6</td></tr>
<tr><td>S protein</td><td>10 µg</td><td>SWE</td><td>6</td></tr>
<tr><td rowspan="7">RBD-L452K-F490W</td><td rowspan="7">2 µg</td><td>None</td><td>6</td></tr>
<tr><td>Alum</td><td>6</td></tr>
<tr><td>SWE</td><td>6</td></tr>
<tr><td>LQ</td><td>6</td></tr>
<tr><td>LMQ</td><td>6</td></tr>
<tr><td>SQ</td><td>6</td></tr>
<tr><td>SMQ</td><td>6</td></tr>
<tr><td>S protein</td><td>2 µg</td><td>SWE</td><td>6</td></tr>
<tr><td rowspan="7">Mouse study #3

Balb/cJ</td><td>RBD</td><td>10 µg</td><td>Alum</td><td>5</td></tr>
<tr><td rowspan="3">RBD-L452K-F490W</td><td rowspan="2">10 µg</td><td>Alum</td><td>5</td></tr>
<tr><td rowspan="2">Alum + CpG</td><td>5</td></tr>
<tr><td>2 µg</td><td>5</td></tr>
<tr><td rowspan="3">RBD-L452K-F490W (VLP)</td><td rowspan="2">2 µg</td><td>Alum</td><td>5</td></tr>
<tr><td rowspan="2">Alum + CpG</td><td>5</td></tr>
<tr><td>0.022 µg</td><td>5</td></tr>
<tr><td rowspan="5">Mouse study #4

Balb/cJ</td><td rowspan="5">RBD-L452K-F490W (VLP)</td><td>5 µg</td><td rowspan="5">Alum + CpG</td><td>5</td></tr>
<tr><td>1.67 µg</td><td>5</td></tr>
<tr><td>0.56 µg</td><td>5</td></tr>
<tr><td>0.19 µg</td><td>5</td></tr>
<tr><td>0.06 µg</td><td>5</td></tr>
<tr><td rowspan="3">Hamster study</td><td>Buffer</td><td></td><td>None</td><td>10</td></tr>
<tr><td rowspan="2">RBD-L452K-F490W (VLP)</td><td rowspan="2">2 µg</td><td>Alum</td><td>10</td></tr>
<tr><td>Alum + CpG</td><td>10</td></tr>
</table>
